# Genoprotective effect of hyaluronic acid against benzalkonium chloride-induced DNA damage in human corneal epithelial cells

**Published:** 2011-12-21

**Authors:** Han Wu, Huina Zhang, Changjun Wang, Yihua Wu, Jiajun Xie, Xiuming Jin, Jun Yang, Juan Ye

**Affiliations:** 1 Eye Center of the 2nd Affiliated Hospital, Medical College of Zhejiang University, Hangzhou, Zhejiang, China; 2 Department of Toxicology, Zhejiang University School of Public Health, Hangzhou, Zhejiang, China; 3 Department of Toxicology, Hangzhou Normal University School of Public Health, Hangzhou, Zhejiang, China

## Abstract

**Purpose:**

The aim of this study was to investigate hyaluronic acid (HA) protection on cultured human corneal epithelial cells (HCEs) against benzalkonium chloride (BAC)-induced DNA damage and intracellular reactive oxygen species (ROS) increase.

**Methods:**

Cells were incubated with different concentrations of BAC with or without the presence of 0.2% HA for 30 min. DNA damage to HCEs was examined by alkaline comet assay and by immunofluorescence microscopic detection of the phosphorylated form of histone variant H2AX (γH2AX) foci. ROS production was assessed by the fluorescent probe, 2',7'-dichlorodihydrofluorescein diacetate (DCFH-DA). Cell apoptosis was determined with annexin V staining by flow cytometry.

**Results:**

HA significantly reduced BAC-induced DNA damage as indicated by the tail length (TL) and tail moment (TM) of alkaline comet assay and by γH2AX foci formation, respectively. Moreover, HA significantly decreased BAC-induced ROS increase and cell apoptosis. However, exposure to HA alone did not produce any significant change in DNA damage, ROS generation, or cell apoptosis.

**Conclusions:**

BAC could induce DNA damage and cell apoptosis in HCEs, probably through increasing oxidative stress. Furthermore, HA was an effective protective agent that had antioxidant properties and could decrease DNA damage and cell apoptosis induced by BAC.

## Introduction

Hyaluronic acid (HA) exists as a high molecular weight biologic polymer of the extracellular matrix, composed of repeating disaccharide units of (β,1–4)-D-glucuronic acid-(β,1–3)-N-acetyl-D-glucosamine [1]. In the eye, HA is abundant in the vitreous body and in low concentrations in the aqueous humor [2]. Among extracellular matrix molecules, HA has unique hygroscopic, rheological, and viscoelastic properties [3]. HA has been used as a tear substitute for dry eyes to increase tear film stability and reduce subjective symptoms, such as ocular irritation and burning [4-6]. It has also been used in ophthalmic practice to protect the corneal endothelium and maintain the anterior chamber depth during intraocular surgery [7,8]. Furthermore, in vitro models have demonstrated that HA might play an important role in corneal epithelial development, wound healing and inflammation [9-13].

Preservatives such as benzalkonium chloride (BAC) are used in most ophthalmic preparations to prevent bacterial contamination. The mechanism of the antimicrobial action of BAC is thought to be due to disruption of the cell membranes of microorganisms. Several studies have confirmed that BAC could enhance drug penetration and improve topical bioavailability of ophthalmic drugs [14-16]. Although topically administered medications are increasingly used with apparent safety and good tolerance, there is growing evidence that long-term use of topical drugs containing BAC may have adverse effects on the corneal epithelium. Many in vivo and in vitro studies have been developed to predict the toxic effects of BAC on corneal and conjunctival epithelium, such as ocular irritation, corneal surface impairment, tear film instability, corneal epithelial barrier dysfunction, cell apoptosis, and the potential risk of failure for future glaucoma surgery [17-21].

In our previous study, we showed that exposure to BAC in human corneal epithelial cells (HCEs) even at low concentrations could induce DNA strand breaks, which were still present after BAC removal [22]. In the current study, we examined whether HA could influence the effects of BAC on HCEs. As reported herein, we found that HA could protect HCEs from the BAC-induced genotoxic effects and ROS formation.

## Methods

### Cell culture

Simian virus (SV) 40-immortalized human corneal epithelial cells (HCEs) [23] were provided by New York University (New York, NY) and were cultured in DMEM/F12 (Gibco, Grand Island, NY), supplemented with 10% fetal bovine serum (Gibco), 5 µg/ml insulin (Gibco), 0.1 µg/ml cholera toxin, 5 ng/ml human epidermal growth factor (Gibco), and 40 µg/ml gentamicin and cultured in 25 cm^2^ cell culture flasks at 37 °C in an atmosphere of 95% air and 5% CO_2_. Confluent cultures were removed by 0.25% trypsin-EDTA (Sigma Aldrich, St. Louis, MO) incubation, and cells were counted, plated on sterile glass coverslips for the phosphorylated form of histone variant H2AX (γH2AX) detection, in six-well plates for alkaline comet assay, reactive oxygen species (ROS), and apoptosis detection.

### Cell treatments

When cells reached approximately 80% confluence, the culture medium was removed. Cells were incubated for 30 min with 0.00005%, 0.0001%, 0.0005%, and 0.001% BAC (BAC/HA^-^), or treated with a combination of 0.2% HA (1,000 kDa; Freda Biopharm Co., Ltd., Shandong, China) and different concentrations of BAC (BAC/HA^+^). BAC and HA were dissolved in culture medium; thus culture medium was used as a negative control.

### DNA damage detection

DNA damage was examined by comet assay and by immunofluorescence microscope detection of γH2AX foci.

#### Comet assay

The alkaline comet assay was performed as previously described with some modiﬁcations [24]. First, the fully frosted microscope slide was covered with 100 μl of 0.65% normal melting point (NMP) agarose and immediately covered with a coverslip. Slides were placed on ice to allow the agarose to solidify. Second, cells were mixed with 0.65% low melting point (LMP) agarose (75 μl) to form an LMP-cell suspension. After putting the coverslip back, the slide was allowed to solidify on ice for several min. Third, another layer of agarose (75 μl of 0.65% LMP agarose) was added as described before. Following slide preparation, the embedded cells were lysed by gently immersing the slides in the freshly prepared ice-cold lysis solution (2.5 M NaCl, 100 mM EDTA, 10 mM Tris, with 1% Triton X-100 and 10% DMSO added just before use, pH 10). After at least 1 h at 4 °C in the dark, the lysis solution was removed, and the slides were rinsed three times with distilled water. The slides were then placed in a horizontal gel electrophoresis chamber filled with fresh buffer (300 mM NaOH, 1 mM EDTA, pH>13) for 20 min to allow DNA to unwind. Electrophoresis was conducted in the same buffer at 20 V and 300 mA for 20 min. Then the slides were washed twice in a neutralization buffer (0.4 M Tris, pH7.5) and fixed in methanol for 3 min. The slides were stained with 20 μg/ml ethidium bromide and observed using an Olympus AX70 fluorescent microscope (Olympus, Tokyo, Japan). The tail length (TL) and tail moment (TM) was measure by ImagePro Plus software (Media Cybernetics, Silver Spring, MD) in at least 100 cells on one slide.

#### Immunofluorescent microscopy and quantification of γH2AX foci

Immunofluorescent microscopy was performed basically the same as described earlier with modifications [25]. In short, after treatment, cells were fixed in 4% paraformaldehyde for 15 min, washed twice with PBS, and permeabilized in 0.2% Triton X-100 (Sigma). After being blocked with 3% blocking serum albumin (Sigma) for 1.5 h, samples were incubated with 1:1,000 mouse monoclonal anti-γH2AX antibody (Upstate Technology, Lake Placid, NY) for 2 h, followed by 1:500 FITC-conjugated goat-anti-mouse secondary antibody (AF488; Invitrogen, Carlsbad, CA) for 1 h. To stain the nuclei, 4',6-diamidino-2-phenylindole (DAPI) was added to the cells and incubated for another 15 min. The coverslip was then removed from the plate, mounted on a glass slide, and observed with an Olympus AX70 fluorescent microscope (Olympus). To prevent bias in selection of cells that display foci, all the cells were counted in the field of vision (at least 50 cells). Image Pro Plus (Media Cybernetics) was used to count the γH2AX foci in each cell.

### Intracellular ROS detection

ROS was measured with membrane permeable dye 2′,7′-dichlorodihydrofluorescein diacetate molecule probes (DCFH-DA; Sigma), using a slight modification of the previously published method [26]. Cells were collected and centrifuged, and supernatant was discarded. The pellet was washed twice with PBS, resuspended in PBS containing a final concentration of 10 μM DCFH-DA. After a 30-min incubation, the cells were centrifuged and washed three times with PBS. After resuspension with PBS, cells were measured immediately, using flow cytometry (Cytomics FC 500; Beckman Coulter Inc., Miami, FL) to monitor the formation of the fluorescent-oxidized derivative of DCFH-DA at an emission wavelength of 525 nm and an excitation wavelength of 488 nm. ROS were detected immediately after incubation to provide reliable data. Statistical analysis was performed using specialized software (CXP software; Beckman Coulter Inc.). For each sample, at least 10,000 events were analyzed in each of three independent experiments. The ROS level was represented as the mean fluorescence intensity (MFI) of DCFH-DA in treated sample/the MFI in control group.

### Flow cytometry analysis of cell apoptosis

The annexin V-ﬂuorescein isothiocyanate (FITC)/propidium iodide (PI) kit (Biovison. Int., Mountain View, CA) was used to assess modifications of the cell membrane that are associated with program med cell death. Experiments were conducted according to the manufacturer’s instructions. In short, after treatment with BAC, cells were collected, counted, centrifuged, and resuspended to 5×10^5^ cells in 500 μl of 1× binding buffer. Annexin V-FITC (5 μl) and 10 μl PI were added to each sample. The samples were incubated in the dark at room temperature for 5 min. Samples were then examined immediately on the Cytomics FC 500 flow cytometry using the CXP software for data analysis. At least 5,000 cells were analyzed in each treatment group.

### Statistical analysis

Each experiment was conducted at least three times. Statistical analysis was performed with the Student *t*-test and one-way ANOVA followed by the Dunnett multiple comparison test (GraphPad Prism 5 software; GraphPad Software, San Diego, CA). Statistically significant differences between groups were considered to have a p value of <0.05. Results were expressed as the mean±standard error of more than three experiments.

## Results

### HA significantly reduced BAC-induced DNA damage detected by comet assay

**Figure 1:**
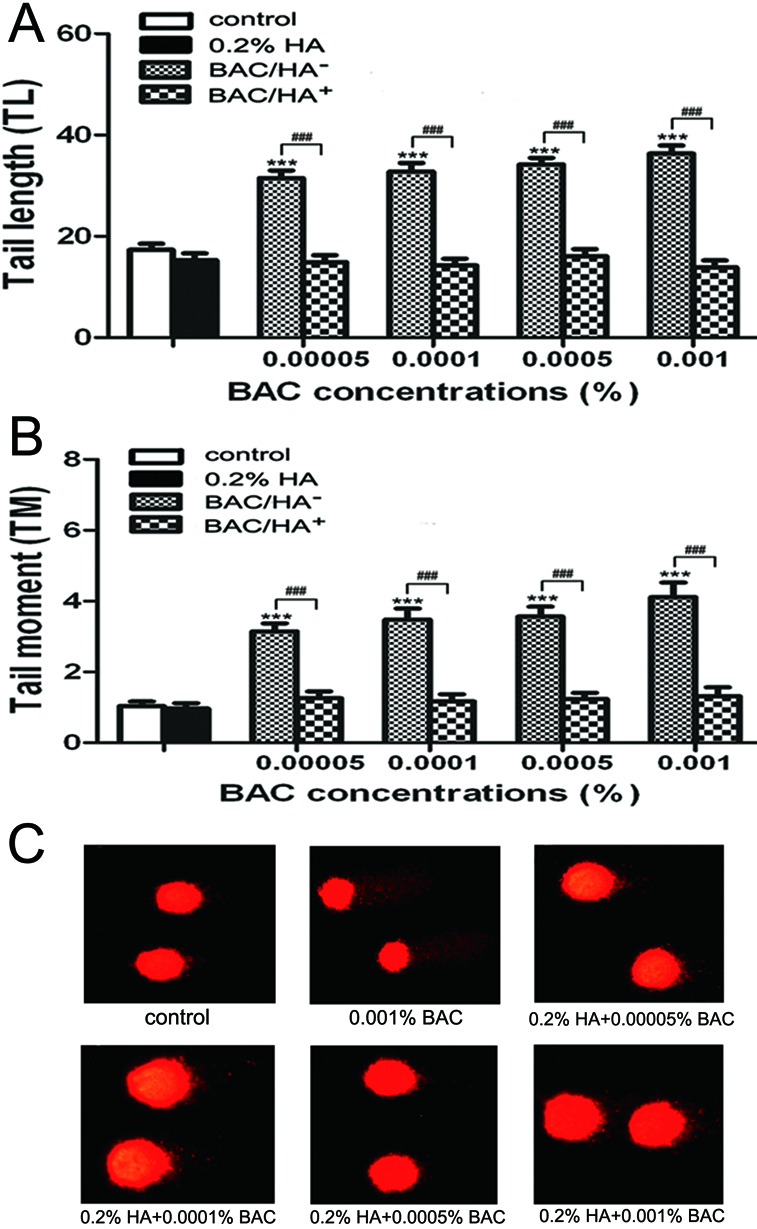
Alkaline comet assay showed that HA could reduce BAC-induced DNA damage. HCEs were treated with four different concentrations of BAC with or without 0.2% HA. **A**: Tail length (TL). **B**: Tail moment (TM). **C**: Representative images of alkaline comet assay. Incubation at various concentrations of BAC for 30 min produced a significant increase in the level of TL and TM, whereas a combination of 0.2% HA and BAC showed a significant decrease. Differences were significant at p<0.001 (three asterisks) compared to control cells, and p<0.001 (three hash marks) compared between cells treated with BAC alone (BAC/HA^-^) and cells treated with a combination of 0.2% HA and BAC (BAC/HA^+^).

In our previous study, we confirmed that BAC could cause DNA single-strand breaks (SSBs) as indicated by Olive tail moment (OTM) of alkaline comet assay [22]. In addition, as shown in Figure 1, it was found that BAC had a clear dose-dependent effect on DNA fragmentation as indicated by the tail length (TL) and tail moment (TM) of alkaline comet assay (p<0.001 compared to the control group). Furthermore, cells treated with a combination of 0.2% HA and BAC showed significantly fewer TM and TL than those treated with BAC alone (Figure 1; p<0.001).

### HA significantly decreased DSBs induced by BAC

**Figure 2:**
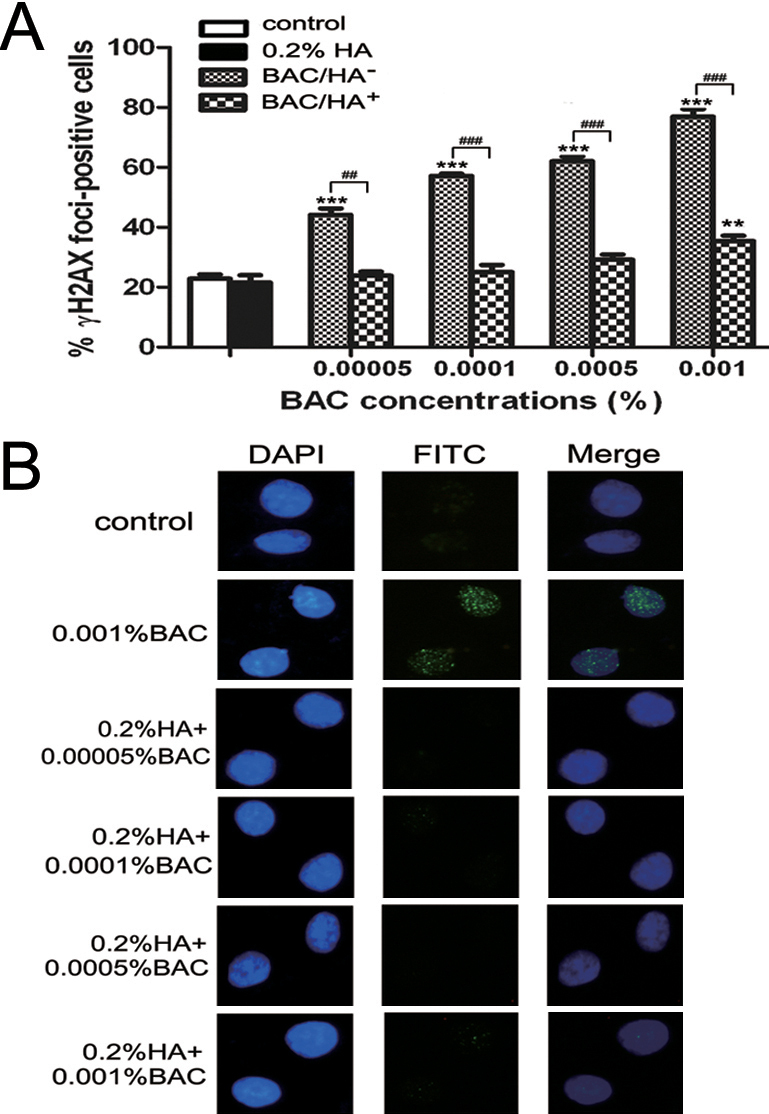
The results of γH2AX foci formation in HCEs after BAC with or without HA treatment. **A**: The percentage of γH2AX foci-positive cells. After 30 min incubation, four concentrations of BAC-treated cells showed a significant increase DSBs compared to the control group, whereas a combination of 0.2% HA and BAC resulted in a significantly smaller number of DSBs. The cells treated with a combination of 0.2% HA and 0.001% BAC showed a significant increase in the percentage of γH2AX foci-positive cells compared to the control cells. **B**: Representative images of γH2AX foci. The nuclei stained by DAPI exhibit in blue, while the γH2AX foci stained by FITC exhibit in green. Differences were significant at p<0.01 (two asterisks) and p<0.001 (three asterisks) compared to control cells, and p<0.01 (two hash marks) and p<0.001 (three hash marks) compared between cells treated with BAC alone (BAC/HA^-^) and cells treated with a combination of 0.2% HA and BAC (BAC/HA^+^).

γH2AX foci, which represent phosphorylation at Ser139 of histone variant H2AX, are used as sensitive biomarkers for DNA double strand breaks (DSBs) [27]. We recently proved that higher concentrations of BAC could yield more γH2AX foci in HCEs [22]. As shown in Figure 2A, the percentages of γH2AX foci-positive cells in four concentrations of BAC-treated cells were significantly greater than those found in the control cells (p<0.001). Cells treated with a combination of 0.2% HA and four concentrations of BAC resulted in a significantly smaller number of γH2AX foci-positive cells compared to BAC-exposed cells without HA incubation (p<0.01; Figure 2A); however, the cells incubated with a combination of 0.2% HA and 0.001% BAC showed a significant increase in the percentage of γH2AX foci-positive cells compared to the control cells (p<0.01; Figure 2A).

### HA significantly decreased BAC-induced oxidative stress

**Figure 3:**
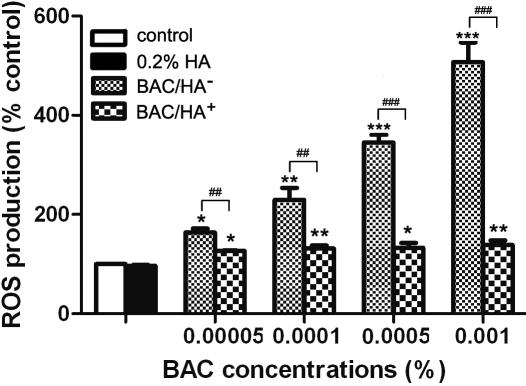
HA significantly decreased BAC-induced oxidative stress. ROS production significantly increased after 30 min of exposure at four concentrations of BAC, which was inhibited by 0.2% HA. However, cells treated with a combination of 0.2% HA and BAC showed a significant increase in ROS production compared to the control cells. Differences were significant at p<0.05 (one asterisk), p<0.01 (two asterisks), and p<0.001 (three asterisks) compared to control cells, and p<0.01 (two hash marks) and p<0.001 (three hash marks) compared between cells treated with BAC alone (BAC/HA^-^) and cells treated with a combination of 0.2% HA and BAC (BAC/HA^+^).

ROS production was represented as the mean fluorescence intensity (MFI) of DCFH-DA in treated sample/the MFI in control group. After incubation for 30 min, there was a dose-dependent increase of ROS production in each BAC/HA^-^-treated group. The MFI of the various concentrations of BAC increased to 163.8%, 229.5%, 345.4%, and 507.0% compared to control groups, respectively, each of which was significantly higher than those found in the control cells (p<0.05; Figure 3). 0.2% HA incubation inhibited the increase in ROS generation induced by BAC (p<0.01; Figure 3); however, cells treated with a combination of 0.2% HA and BAC showed a significant increase in ROS production compared to the control cells (p<0.05; Figure 3).

### HA significantly decreased BAC-induced cell apoptosis

**Figure 4:**
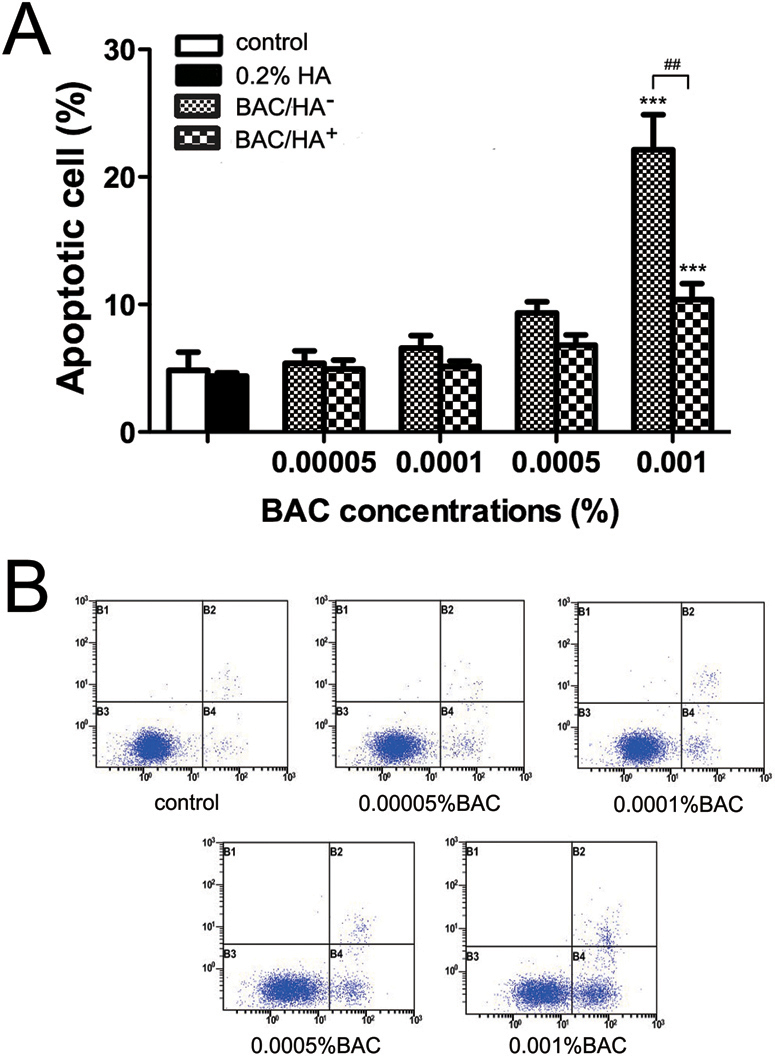
HA significantly decreased BAC-induced cell apoptosis. **A**: No significant differences in apoptosis of HCEs cells were found at concentrations ranging from 0.00005% to 0.0005%. 0.001% BAC showed a significant increase in cell apoptosis, whereas 0.2% HA was significantly decreased 0.001% BAC-induced cell apoptosis. Cell apoptosis in 0.2% HA and 0.001% BAC co-incubated group showed a significantly increase compared to the control cells. **B**: Representative images of cell apoptosis examined by the flow cytometry. Differences were significant at p<0.001 (three asterisks) compared to control cells, and p<0.01 (two hash marks) compared between cells treated with BAC alone (BAC/HA^-^) and cells treated with a combination of 0.2% HA and BAC (BAC/HA^+^).

After 30 min incubation with BAC, no significant differences in apoptosis of HCEs were found at concentrations ranging from 0.00005% to 0.0005% (p>0.05 compared to the control, Figure 4). At a concentration of 0.001%, the percentage of apoptotic cells was 22.1% as compared with the control group (4.8%), p<0.001, which represented a significant increase. 0.2% HA significantly decreased 0.001% BAC-induced cell apoptosis (p<0.01; Figure 4); however, the percentage of apoptotic cells in 0.001% BAC/HA^+^-treated group showed a significant increase compared to the control cells (p<0.001; Figure 4).

## Discussion

BAC is commonly used as a preservative in most ocular medications, and the most common concentration of BAC used in eye drops ranges from 0.001% to 0.1%. In accordance with previous in vitro studies [19,28-30], we confirmed that BAC was a toxic agent. The objective of this study was to investigate in vitro whether HA could protect human corneal epithelial cells from the genotoxic effects of BAC treatment.

The alkaline comet assay is a sensitive method for direct visualization of DNA single-strand breaks (SSBs) on the level of a single cell [31]. γH2AX foci formation has been suggested as another specific and sensitive indicator for DNA double-strand breaks (DSBs) [32]. Moreover, the disappearance of γH2AX foci is associated with the complication of DSBs repair [33]. The results of our alkaline comet assay demonstrated that BAC had a clear dose-dependent effect on DNA fragmentation as indicated by the tail length (TL) and tail moment (TM) (Figure 1). BAC-induced DSBs, detected by γH2AX immunofluorescent staining, were also observed in HCEs in a dose-dependent manner (Figure 2). In the present study, HA effectively reduced the SSBs and DSBs induced by BAC in HCEs, demonstrating a genoprotective effect.

Oxidative stress has been recognized as one of the main causes of DNA damage. It is well known that reactive oxygen species (ROS) are generated as a by-product of normal mitochondrial activity in aerobic cells. ROS overproduction can cause severe damage to cellular macromolecules, especially the DNA [34]. Experimental studies revealed that in vitro many extracellular stimuli could influence the redox cycling pathway, causing the formation of ROS and eventually leading to SSBs and DSBs [35,36]. Debbascb et al. [37] proposed that oxidative stress might play an important role in tissue damage induced by BAC in ocular surface disorders. In our study, a dose-related increase in ROS levels was also observed in HCEs exposed to various concentrations of BAC (Figure 3). It was possible that the surplus ROS produced by BAC disturbed the balance between the oxidation and reduction systems, eventually leading to DNA damage.

Preliminary in vitro studies have demonstrated that BAC could induce arrest of cellular growth and cell death on both corneal and conjunctival epithelial cells [20,29]. We also observed a significantly increased apoptosis in HCEs after 30 min of treatment with BAC 0.001% (Figure 4). It has been reported that DNA damage can have dramatic effects on cell cycle arrest, apoptosis, or oncogenesis [38,39]. In addition, Buttke et al. [40] confirmed that excessive levels of ROS could induce apoptosis in various cell types. Therefore, we supposed that higher concentrations of BAC could cause overproduction of ROS, which might influence the cellular viability by inducing more SSBs and DSBs.

HA is a remarkable biopolymer that appears to have an impressive array of biologic functions. Human studies have confirmed that HA could increase tear film stability and reduce subjective symptoms of dry eyes, such as ocular irritation and burning [4-6]. Furthermore, several experiments in animals have shown that HA could promote corneal epithelial wound healing by stimulating the migration, adhesion, and proliferation of the corneal epithelium [9,41]. In vitro models have also demonstrated that HA could protect cells against cell death, inflammation, and oxidative stress in ocular surface epithelial cells [13,42]. In our present study, we demonstrated that exposure to HA alone did not induce any toxicity in HCEs, which was consistent with the study of Pauloin et al. [43]. We also observed that HA possessed antioxidant and anti-apoptotic properties. Results showed that cell apoptosis and oxidative stress was significantly lower in 0.2% HA and BAC co-incubated cells than those treated with BAC alone (Figure 3 and Figure 4). In addition, alkaline comet and γH2AX foci assays showed that HA effectively reduced the SSBs and DSBs induced by BAC in HCEs (Figure 1 and Figure 2). These results lead to the conclusion that HA had a significant protective effect. A possible explanation was that HA with negative charges could neutralize the toxic effect caused by the cationic charge of the BAC quaternary ammoniums to the corneal epithelial cells [42]. Another hypothesis was that the viscous biopolymer formed a protective coat on cell membrane by binding to specific cell-surface receptors including CD44, which was demonstrated to be expressed in the corneal epithelial cells [43], thus decreasing the interaction between the toxic agents and the cells. Furthermore, HA is rich in hydroxyl functions that can potentially absorb ROS [43]. Thus, we suggested that HA indirectly prevented BAC-induced DNA strand breaks and cell apoptosis in HCEs by decreasing ROS production.

In conclusion, our data showed that the preservative BAC could cause intracellular ROS overproduction, and cause DNA strand breaks and cell death in HCEs even at low concentrations. In addition, HA, which had no toxic effect on HCEs, could significantly reduce all the BAC-induced toxic effects we observed. We suggested that HA was an effective protective agent that had antioxidant properties and could decrease DNA damage and cell apoptosis induced by BAC. In the future, a considerable improvement of ocular tolerance may be obtained by adding HA to preserved ophthalmic drugs. However, the experiments conducted in vitro may not reflect the real situation in vivo. Therefore, further investigation is still needed to confirm the significance of these findings in vivo.
